# Direct analysis of tobacco specific nitrosamines in tobacco products using a molecularly imprinted polymer-packed column

**DOI:** 10.3389/frans.2022.1091206

**Published:** 2023-01-06

**Authors:** Haley A. Mulder, Justin L. Poklis, Adam C. Pearcy, Matthew S. Halquist

**Affiliations:** 1 Department of Pharmaceutics, School of Pharmacy, Virginia Commonwealth University, Richmond, VA, United States; 2 Department of Pharmacology and Toxicology, School of Medicine, Virginia Commonwealth University, Richmond, VA, United States

**Keywords:** direct analysis, tobacco specific nitrosamines, eliquids, nicotine and tobacco products, molecularly imprinted polymer (MIP)

## Abstract

Tobacco specific nitrosamines (TSNAs) are highly carcinogenic by-products in tobacco samples, and their presence is regulated by the Food and Drug Administration. Molecularly imprinted polymers (MIPs) are synthetic polymers that have been “imprinted” with a template analyte in a co-polymer system, and can selectively extract analytes from complex matrices. MIPs can be incorporated into online systems, replacing traditional high performance liquid chromatography (HPLC) columns. MIP material specific for TSNAs was packed into an empty HPLC column using a slurry packing technique. The developed method with the MIP-packed HPLC column was validated on a LC-MS/MS system for the quantitation of N-nitrosonornicotine (NNN) and 4- (methylnitrosamino)-1-(3-pyridyl)-1-butanone (NNK) in commercial tobacco products. The method was linear over .1–10 ng/ml (.4–10 μg/g) for NNN and NNK. The limit of detection (LOD) was .03 ng/ml (12 μg/g) and the limit of quantitation (LOQ), .1 ng/ml (.4 μg/g). All column uniformity parameters with the exception of theoretical plate number were within the accepted criteria (% RSD values <15%). Theoretical plate number was <250, owing to the large (50 μm) sized MIP particles. Twenty-six tobacco products contained TSNA concentrations that were consistent with reported literature values. The TSNA-MIP based HPLC column effectively replaced a traditional reverse phase HPLC column, and was used for the direct analysis of nicotine and tobacco products without extensive sample preparation prior to instrumental analysis.

## 1 Introduction

Tobacco specific nitrosamines (TSNAs) are a class of compounds within the tobacco family that are by-products formed from the harvesting, curing, and fermenting of tobacco products (Hecht and Hoffmann, 1988). N-nitrosonornicotine (NNN) and 4-(methylnitrosamino)-1-(3-pyridyl)-1-butanone (NNK) are TSNAs that are carcinogenic, contributing to mouth, throat, and lung cancer in association with cigarette use, and are listed on the FDA’s Harmful and Potentially Harmful Constituents list (Konstantinou et al., 2018; Holman, 2019). The Food and Drug Administration (FDA) has proposed recommendations that NNN levels in smokeless tobacco be limited to 1 μg/g. (U.S. Food and Drug Administration, 2017). The FDA, however, does not have any current recommendations TSNA levels in electronic cigarettes (E-cigarettes). While e-cigarettes have been marketed as a safer alternative to cigarettes, both NNN and NNK have been reported as being detected in e-cigarette e-liquids and e-cigarette aerosols (Farsalinos et al., 2015; Flora et al., 2016; Goniewicz et al., 2014). Levels for TSNAs in e-liquids have been reported from .22 to 9.84 ng/ml for NNN and .11–1.11 ng/ml for NNK respectively, which is lower than the TSNA levels found in traditional cigarettes (Goniewicz et al., 2014; Kim and Shin, 2013).

Since TSNAs are found at low levels inside nicotine and tobacco products, and at even lower concentrations in e-cigarette products, sensitive and selective methods are required for TSNA analysis. Current analytical methods for the extraction and detection of TSNAs in smokeless tobacco products require multiple extraction methods, such as liquid-liquid extraction followed by SPE extraction (Pang et al., 2019). The use of solid phase extraction (SPE) for the extraction of TSNAs in e-liquids, however, report less than 30% recovery with various methods (Kim and Shin, 2013). The extensive sample preparation to achieve desirable recoveries results are time consuming, and are ultimately not specific for the extraction of TSNAs.

Molecularly imprinted polymers (MIPs), are an alternative sample preparation tool. MIPs are synthetic polymers that have been “imprinted” with a specific analyte, allowing for selective extractions in complex matrices (Hu et al., 2021). The analyte of interest can fit into the imprinted cavity and selectively interact with a functional monomer through non-covalent interactions (electrostatic interactions, hydrogen bonding, etc., ). The specific selectivity of the imprinted polymer for the analyte of interest allows cleaner sample extracts, resulting in better detection and higher recoveries of the sample with analytical instruments. MIPs are most commonly used in place of supports for SPE cartridges. Commercial MIP SPE cartridges are available, such as the SupelMIP SPE cartridges for TSNAs (Boyd et al., 2007; Kavvadias et al., 2009; Mulder HA, 2020).

MIPs can also be used to replace traditional HPLC columns, allowing for the sample to be simultaneously extracted and separated on the instrument, prior to introduction to the analytical detector (Baykara et al., 2012; Figueiredo et al., 2011; Santos et al., 2017; Sellergren, 1994; Shah et al., 2011). While the analyte is retained inside the imprinted cavity, interferents and matrix components only interact with the surface of the polymer through non-specific interactions. Through careful selection of solvents, the interferents can be washed off the polymer and directed to waste before the analyte is eluted and directed to the detector. Currently, there are no commercial MIP-HPLC based columns, requiring in-house preparations. This is achieved by polymerizing monolithic stationary phases inside the analytical column, or mixing polymer particles with a solvent to create a slurry that is then packed into the analytical column under high, constant pressure (Sambe et al., 2006; Sellergren, 1994; Shah et al., 2011).

Presented is the development of a molecularly imprinted high performance liquid chromatography (MIP-HPLC) column by a slurry packing method using the commercial TSNA imprinted polymer for the direct analysis of TSNAs in nicotine and tobacco products. The analytical method using the MIP-packed HPLC column was validated following the proposed guidelines from the US Department of Health and Human Services Center for Tobacco Products Guidance for Industry (U.S. Food and Drug Administration, 2021). The MIP-packed column was also characterized for chromatographic properties and column uniformity of the packing method. Nicotine and tobacco products consisting of moist oral snuff (SNUS), oral nicotine products, pipe tobacco, and e-cigarette e-liquids were analyzed using the MIP-HPLC column.

## 2 Materials and methods

### 2.1 Materials and chemical reagents

TSNA molecularly imprinted polymer powder (particle size 30–90 μm) was purchased from Biotage (Uppsala, Sweden). Acetic acid, ammonium hydroxide (28%–30%), HPLC grade acetonitrile, chloroform, isopropyl alcohol (IPA), methanol, toluene, and water were purchased from VWR International (Radnor, PA). Ethanol (EtOH) and formic acid were purchased from Sigma Aldrich (St. Louis, MO). N-nitrosonirnicotine (NNN), NNN-d4, and 4-(Methylnitrosamino)-1-(3-pyridyl)-1-butanone (NNK) were purchased from Toronto Research Chemicals (Toronto, ON). Propylene glycol (PG) was purchased from Amresco LLC, VWR, United States. USP grade vegetable glycerin (VG) was purchased from JT Baker, United States.

### 2.2 Preparation of reagents

Standard stock solutions of NNN (1 mg/ml), NNK (1 mg/ml) and NNN-d4 (100 μg/ml) in methanol were used for analysis. Working stock concentrations of 1,000 and 100 ng/ml of NNN/NNK in methanol were stored at −20°C. Working stock concentration of 10 μg/ml NNN-d4 was prepared in methanol and stored at −20°C. Calibrators were prepared fresh, daily in 10 mM ammonium acetate, pH 5.5. The internal standard, NNN-d4, was prepared daily at 25 ng/ml in 10 mM ammonium acetate, pH 5.5. Quality control (QC) samples in a matrix to match the SNUS, oral nicotine products, and pipe tobacco were prepared in 10 mM ammonium acetate, pH 5.5. Quality control samples in a matrix to match electronic cigarettes e-liquids were prepared by dissolving NNN/NNK working standards in a mixture of propylene glycol and vegetable glycerin (70:30 v/v). Prior to analysis, e-liquid QCs were diluted 1:10 in 10 mM ammonium acetate, pH 5.5.

### 2.3 Preparation of TSNA MIP column

**FIGURE 1:**
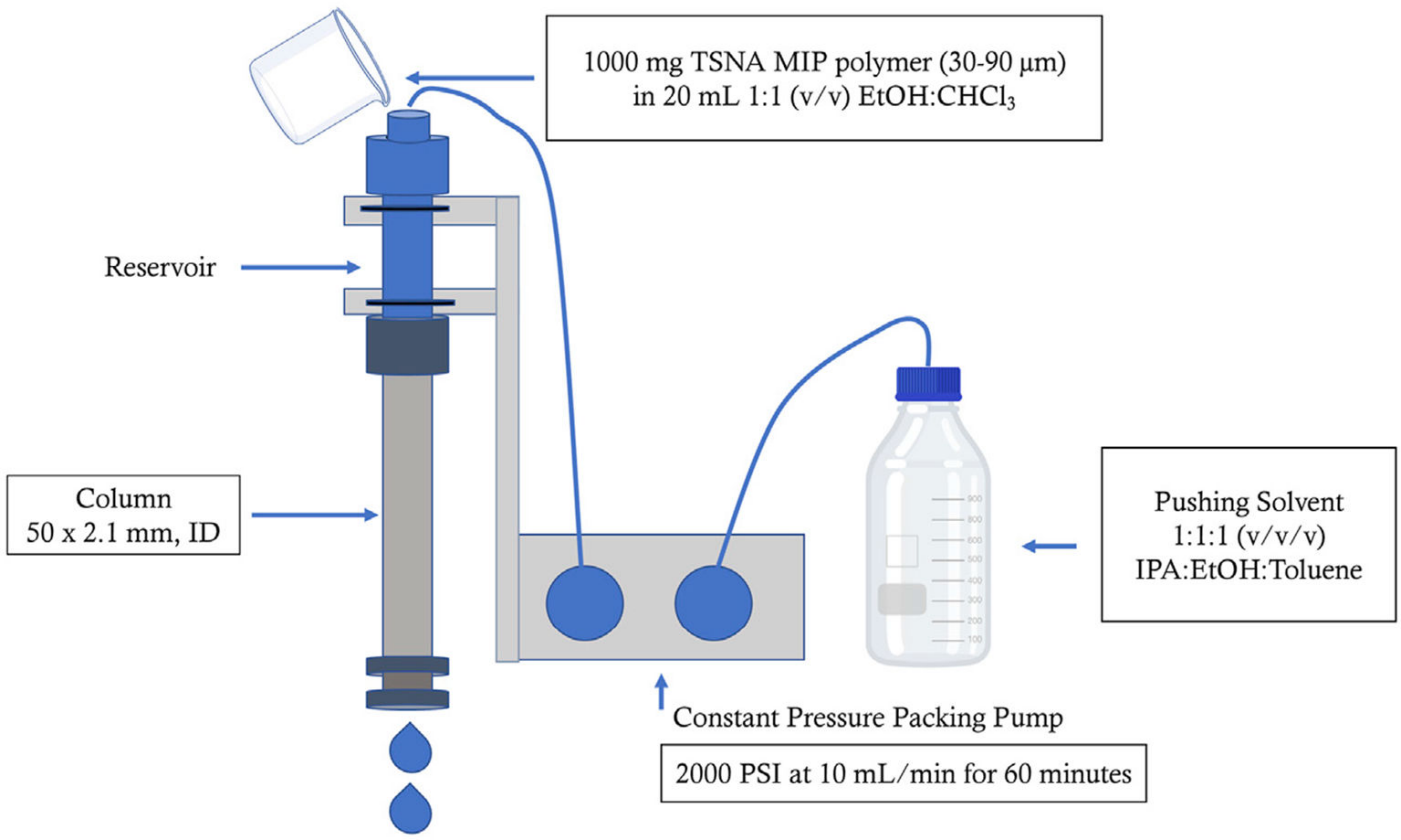
Preparation of TSNA MIP HPLC packed column. An empty 50 × 2.1 mm, ID Restek column was attached to a reservoir on a Teledyne Constant Pressure Packing Pump. A slurry mixture consisting of 1,000 mg MIP mixed with 20 ml of 1:1 (v/v) ethanol:chloroform was poured into the reservoir. The slurry was pushed through the reservoir and into the column with 1:1:1 (v/v/v) ethanol:isopropyl alcohol:toluene operated a 10 ml/min (2000 PSI) for 1 hour.

The TSNA MIP column was prepared following a modified method (Forzano et al., 2019) under slurry conditions using a Teledyne (Thousand Oaks, CA) packing pressure system. The end fitting and .2 μm frit from one end of a Restek (Bellefonte, PA) column assembly kit (50 mm × 2.1 mm ID, ¼” OD) was removed and attached to a reservoir with a glass frit (Figure 1). One Gram of TSNA MIP material from Biotage was suspended in 20 ml of a 1:1 (v/v) chloroform:ethanol solution to create a slurry, which was sonicated for 15 s. The mixture was poured into the reservoir and the system was capped and secured. Pushing solvent consisting of 1:1:1 (v/v/v) ethanol:isopropyl alcohol: toluene was used to push the slurry mixture through the column. Helium gas was used to degas the solvent during the packing process. The pushing solvent was slowly increased from .5 ml/min to 10 ml/min with a total system pressure around 2000 psi. The flow rate of the pushing solvent was kept at 10 ml/min for 1 hour before the system was disassembled and the previously removed frit and end fitting were reassembled on the column. The freshly packed column was washed with 100% acetonitrile at a flow rate of .5 ml/min for 60 min using an external Shimadzu LC-10AD HPLC pump (Kyoto, Japan). Three HPLC columns were packed with TSNA MIP material from the same lot.

### 2.4 Chromatographic conditions

Method development and validation was carried out using a SCIEX ExionLC 2.0 Binary Pump UPLC equipped with a SCIEX SelexION 6,500 + Q-Trap. Mobile phase A was 10 mM ammonium acetate, pH 5.5 and mobile phase B was .1% formic acid in methanol. The in-house MIP-packed column (50 × 2.1 mm ID, 30–90 μm) was operated at a flow rate of .45 ml/min at a temperature of 40°C. The autosampler was kept at a temperature of 5°C, and the injection volume was 10 μL. A gradient method was developed and outlined in Table 1. The entire analytical run time was 5 minutes. The ESI source of the mass spectrometer was operated in positive mode. The declustering potential was set to 20 eV and the temperature of the source was set to 550°C. Ion source gases one and two were set to 60 and 25 ml/min, respectively. The mass spectrometer was operated in multiple reaction monitoring mode (MRM) for the following ions with collision energy in parentheses: NNN, 178 > 120 m/*z* (27 V) and 178 > 148 m/*z* (14 V); NNK, 208 > 122 m/*z* (16 V) and 208 > 148 m/*z* (18 V); and NNN-d4, 182 > 124 m/*z* (27 V) and 182 > 152 m/*z* (14 V).

### 2.5 Method validation

Method validation was carried out following the US Department of Health and Human Services Center for Tobacco Products Guidance for Industry for linearity, limit of quantitation (LOQ), and accuracy and precision (U.S. Food and Drug Administration, 2021). Column uniformity and autosampler stability were also assessed during this method validation. Six calibration standards having concentrations of .10, .25, .50, 1.00, 5.00, and 10.0 ng/ml (.04–10 μg/g) of NNN and NNK were prepared in 10 mM ammonium acetate, pH 5.5 in triplicate. Linearity was evaluated through linear regression with a weighting of 1/x and coefficient of variation (*r*^*2*^). Standards were back-calculated from the generated linear regression. The residual concentrations of the back-calculated values, known as amount deviation from normal (%DFN), should not exceed 15%. The lower limit of quantitation (LLOQ) was the lowest calibration standard concentration of NNN and NNK. Accuracy and precision were determined from quality control (QC) samples injected in triplicate for three different validation runs (*N* = 9). QC samples prepared in 10 mM ammonium acetate, pH 5.5 were prepared at four concentrations: A limit of quantitation-QC (LLQC, .1 ng/ml), a low-QC (LQC, .3 ng/ml), a medium-QC (MQC, 3.0 ng/ml), and a high-QC (HQC, 7.5 ng/ml). QC samples prepared in 70: 30 (v/v) PG:VG were diluted 1:10 to three concentrations: A low-QC (LQC, .3 ng/ml), a medium-QC (MQC, 3.0 ng/ml), and a high-QC (HQC, 7.5 ng/ml). Accuracy for calculated concentration of the quality controls should not exceed ± 15% of the nominal concentrations (%DFN). Precision was expressed as a percent relative standard deviation (%RSD) and should not exceed 15%.

### 2.6 Autosampler stability

Autosampler stability was determined by reinjecting a set of controls that were left in the autosampler for 24 h, 48 h, and 72 h. The calculated concentrations were compared to a fresh set of controls, prepared daily, alongside each injection set. Acceptable criteria for autosampler stability were accuracy values within ±15% of the nominal concentrations (%DFN) and precision levels calculated as %RSD not exceeding 15%.

### 2.7 Column uniformity

Three HPLC columns were packed on separate occasions following the methods outlined in section 2.3. Low, medium, and high QC concentrations in 10 mM ammonium acetate, pH 5.5, and 70:30 PG:VG were injected in triplicate onto each column. Retention time, peak area, calculated concentration, accuracy, asymmetry, peak tailing, and theoretical plate number (N) were assessed. Tailing factor ($T_{f}$) was calculated as

(1) $$T_{f}=\frac{a+b}{2a}$$

Where a is the front half of the peak at 5% of the peak height and b is the back half of the peak at 5% peak height. Theoretical plate number (N) was calculated as

(2) $$N=5.54{\left(\frac{t_{R}}{W_{50}}\right)}^{{}^{{}^{{}^{2}}}}$$

Where $t_{r}$ is the retention time and $W_{50}$ is the width of the peak at 50% peak height. Acceptable criteria for column uniformity were precision (%RSD) values <15% for each parameter, and calculated concentrations within ± 15% of the nominal value (%DFN).

### 2.8 Preparation of samples

Fourteen electronic cigarette e-liquids were analyzed for this study (Supplementary Figure S1). Ten e-liquids were purchased from various shops in the United States prior to 2016 and stored at room temperature, away from light. Four e-liquids were purchased from shops in Europe after 2016 and stored in the refrigerator. E-liquid samples were prepared in a 1: 5 dilution in 10 mM ammonium acetate, pH 5.5 prior to analysis.

**FIGURE 2:**
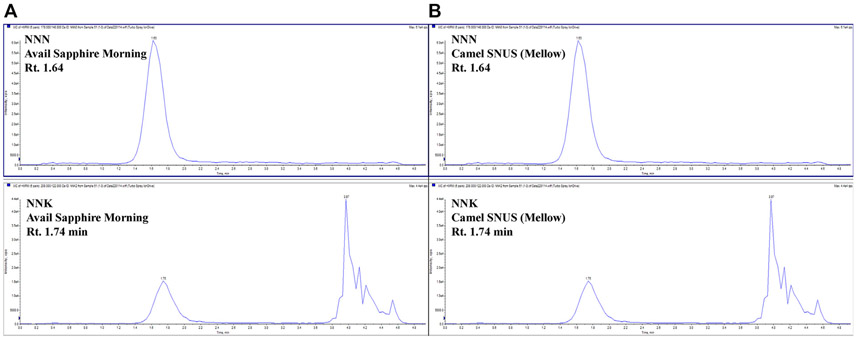
Representative chromatographs of **(A)** an e-liquid sample NNN (Rt. 1.64 min) and NNK (Rt. 1.74 min), and **(B)**, a SNUS product containing NNN (Rt. 1.64 min) and NNK (Rt. 1.74 min) with the TSNA MIP column.

Twelve nicotine/tobacco products described as either oral nicotine pouches, smokeless tobacco (SNUS), or pipe tobacco (labelled dohka) were purchased from various vendors (Figure 2). Samples were stored in their original containers at room temperature, away from light. Samples were prepared following the Cooperation Centre for Scientific Research Relative to Tobacco (CORESTA) method. In brief, 250 mg of sample was removed from the product pouch and vortexed with 10 ml of 10 mM ammonium acetate, pH 5.5 for 60 min (U.S. Food and Drug Administration, 2017). The samples were filtered through a Whatman^™^ .45 μm polyether sulfone membrane syringe (Maidstone, United Kingdom). The SNUS and pipe tobacco products were further diluted in a 1:100 or 1: 1,000 dilution in 10 mM ammonium acetate, pH 5.5.

## 3 Results

### 3.1 Method validation

The method for NNN and NNK was validated following US Department of Health and Human Services Center for Tobacco Products Guidance for Industry. The method developed with the MIP-packed HPLC column had linearity (*r*^2^ > .9985) over six non-zero concentration points from .1 ng/ml to 10 ng/ml (.04 μg/g to 10 μg/g). Accuracy for the six points was between 92.4% and 110% and precision was between 2.5% and 8.2%. The limit of quantitation (LOQ) was .1 ng/ml (.4 μg/g) and the limit of detection (LOD) was .03 ng/ml (.12 μg/g). Table 2 shows the results of the accuracy and precision studies for the quality control (QC) samples. Accuracy for the four QC samples prepared in 10 mM ammonium acetate, pH 5.5, representing SNUS, oral nicotine products, and pipe tobacco were between 91.8% and 97.8% for NNN and 86.5%–99.2% for NNK. Accuracy for the three QC samples representing e-liquid formulations were between 100.5% and 103.9% for NNN and 100.6%–109.7% for NNK. Precision, expressed as %RSD, were <15% for all concentrations of NNN and NNK in all tobacco products. Autosampler stability for NNN and NNK in both solutions indicated stability up to 72 h (Supplementary Figures S2, S3).

### 3.2 Column uniformity and characterization

Three MIP-packed HPLC columns were created over the course of this study. Column uniformity and column characterization was achieved by injecting the quality control calibrators for NNN and NNK in both mediums onto each column in triplicate (*N* = 9). The columns were considered uniform if each parameter had a %RSD value less than 15%. Table 3 describes the results of column uniformity, expressed as retention time (min), peak area, concentration (ng/ml), and accuracy for NNN and NNK respectively. All four parameters had %RSD values less than 15%. Column characterization was expressed as asymmetry, tailing factor, and theoretical plate number (N) in Table 4. Asymmetry values for NNN and NNK were between .9 and 2.2 in both sample mediums and tailing factor values for NNN and NNK were between 1.0 and 1.6. NNK in 70: 30 PG:VG had a %RSD value greater than 15%. Theoretical plate number (N) for NNN and NNK was between 91 and 317 and was highly variable between columns (%RSD >15%).

### 3.3 TSNA content in nicotine E-liquids and tobacco products

The concentrations of NNN and NNK in the e-liquids and tobacco products are reported in Table 5. The Camel SNUS products had between 1.3 and 1.6 μg/g NNN and .44–.45 μg/g NNK present in the three products. The pipe tobacco sample had similar low levels NNN present in the sample, with an average concentration of 1.87 ± .04 μg/g. NNK was not detected in the pipe tobacco. TSNAs were not detected in the oral nicotine samples. In the electronic cigarette e-liquids, reported concentrations for NNN and NNK were 2.1–202.6 ng/ml and 1.5–52.8 ng/ml, respectively.

## 4 Discussion

### 4.1 Method development and characterization of MIP-Packed HPLC column

In this study, a commercial polymer was used to demonstrate the use of molecularly imprinted polymer in the place of a traditional reverse-phase HPLC packing material. Previous studies within this research group characterized NNN’s extraction recovery with a benchtop MIP-SPE cartridge in water and human urine. The highest recoveries for NNN were observed with the TSNA MIP in comparison to a non-imprinted polymer (NIP) and traditional SPE cartridges (Mulder et al., 2022). Using the developed and validated method for the inline MIP HPLC column, the extraction and separation of analytes was achieved *simultaneously* on the analytical instrument, removing the need for extensive benchtop preparation. Further, the MIP-packed HPLC columns were used for over 400 injections without the loss of performance on the column.

The theory behind an in-line MIP column is similar to the interactions of a MIP-based SPE cartridge. In solid phase extraction, the analyte of interest is bound to the stationary phase of the cartridge, and interferents and matrix components are removed through a series of solvent washes (Turiel and Esteban, 2020). When developing an in-line MIP method, the selection of mobile phase solvents is critical for first retaining the analyte on the polymer, and later removing the analyte from the polymer once it has been successfully cleaned of interferents. The starting mobile phase acts as the loading condition in the solid phase extraction, and the subsequent mobile phases are used as washing and/or eluting solvents. It is critical that the starting mobile phase should not prematurely elute the analyte from the column. Following the manufacturer’s recommendations for the SupelMIP TSNA cartridges, which states that samples should be adjusted to a pH of 5.5 prior to loading, the starting mobile phase was 10 mM ammonium acetate, pH 5.5 (“SupelMIP SPE-TSNA Data Sheet,” n.d.). The gradient was increased to 60% mobile phase B, .1% formic acid in methanol, to disrupt the non-covalent interaction between the analytes and the functional monomer. After the analytes were displaced from the column, the gradient was increased to 100% mobile phase B. This was to ensure full removal of the analytes, and to wash the column prior to the next sample. Increasing the temperature of the column from room temperature to 45°C further improved the peak signal. Under these conditions, the entire chromatographic run time was 5 minutes, and NNN eluted at 1.64 min, and NNK eluted at 1.76 min (Figure 2). Furthermore, the only sample preparation required for the nicotine and tobacco products was a simple dilution to fit within the analytical range of the developed method. The method was successfully validated following the proposed guidelines.

As the MIP-packed HPLC columns were made in-house, the reproducibility of the packing method and the performance of the MIP polymer were assessed. Column uniformity was determined by assessing the retention time (min), peak area, concentration (ng/ml), and accuracy of NNN and NNK quality control samples. The columns were considered uniform if each parameter had precision values (Expressed as %RSD) less than 15%. Based on the results for retention time, peak area, calculated concentration, and accuracy (Table 3), the column packing procedure used in this study was able to successfully create multiple, uniform MIP-packed HPLC columns for the purpose of TSNA analysis.

Asymmetry and peak tailing are used to describe the chromatographic peak shape of an analyte. The United States Pharmacopeia (USP) recommends that chromatographic peaks have asymmetry values between .9 and 1.2, and peak tailing less than two ([621] CHROMATOGRAPHY, 2012). The MIP-based HPLC column was slightly asymmetrical with peak tailing that was within acceptable limits (Figure 2; Table 4). The slight peak tailing is most likely from the slow kinetics of the non-covalent interactions between the analyte and functional monomer, which has been previously described in literature (Niu et al., 2016).

Theoretical plate number (N) is used to describe the efficiency of the column in regards to resolution. USP guidance typically recommends that the theoretical plate number be greater than 2000 ([621] CHROMATOGRAPHY, 2012). The theoretical plate number with the MIP columns used in this study were between 200 and 237 theoretical plates and had %RSD values greater than 15% (Table 3). This is 1/10th the recommended theoretical plate number by USP standards. The low theoretical plate number is a result of the large polymer size of the MIP material used in the HPLC column, as theoretical plate number is inversely proportional to particle size. The smaller the particle size, the larger the number of theoretical plates, and therefore the greater the efficiency of the column (Snyder et al., 1997).The polymers used in this study were initially designed for solid phase extraction (SPE), and ranged from 30 to 90 μm in size, with an average polymer size of 50 μm. This is much larger than the traditional RP HPLC polymer size, which are typically 5 μm or smaller. Due to the large particle size of the MIP material, NNN and NNK could not be separated chromatographically. Despite the fact that theoretical plate number did not meet the acceptance criteria for column uniformity, all other parameters in this study met the acceptance criteria and the columns were uniformly packed. Further, column efficiency was not a significant parameter for the analysis of TSNAs with this particular polymer, as they all have different transition ions, allowing for separation within the detector.

The MIP HPLC column and validated method was compared to an established method used for the measurement of NNN and NNK with a traditional, reverse phase C18 column (Zorbax XDB C18, 2.1 × 50 mm, 5 μm). Under similar chromatographic conditions, the C18 column had poor retention (Less than 1 minute) compared with the MIP column (Supplementary Figure S4). Further, the MIP column had better a better signal response in comparison to the C18 column. Compared at .1 ng/ml, the MIP column had a signal response over 100 fold greater for NNK compared to the signal response for NNK on the C18 column. Due to the selective nature of the MIP column’s imprinted cavities, the MIP HPLC column performed better than the traditional reverse phase column in terms of response, accuracy, and had better retention of the tobacco specific nitrosamines.

### 4.2 TSNA content in nicotine E-liquids and tobacco products

The federal registrar has proposed recommendations that oral tobacco products, such as SNUS, have a limit of 1 μg/g of NNN present in products (U.S. Food and Drug Administration, 2017). Using the developed, validated method, it was established that the MIP-HPLC column detected TSNAs present in the SNUS and tobacco samples were within the same range as TSNA levels detected with traditional HPLC columns reported in literature (Lawler et al., 2020). Furthermore, the three Camel SNUS products showed little variation (%RSD <10%) in the amount of NNN and NNK present. The pipe tobacco sample also had low levels of TSNAs present, with an average concentration of 1.87 ± .04 μg/g NNN, and no NNK detected in the sample. The oral nicotine samples contained no presence of TSNAs. The description of the On! and Zyn oral nicotine pouches specifically state that the nicotine in the products are tobacco-free. Therefore, no TSNAs should be present in any of the oral nicotine samples that claim to be tobacco-free.

Compared to the tobacco products, the electronic cigarette e-liquids had a wide distribution of NNN and NNK levels (Table 5). However, the TSNA levels detected with the MIP-HPLC column were within the same reported ranges as TSNAs in e-liquids detected with traditional HPLC columns. While e-cigarettes have been marketed as a safer alternative to traditional cigarettes, TSNAs are still present in the e-liquids and are capable of being inhaled by e-cigarette users (Farsalinos et al., 2015; Goniewicz et al., 2014; Gunduz et al., 2016). Both NNN and NNK are listed on the FDA’s Harmful and Potentially Harmful constituents list, and are noted to be the leading causes of mouth, throat, and lung cancer in association with cigarette use (Hecht and Hoffmann, 1988). Most e-liquid labels do not disclose if the nicotine content is derived from tobacco, and there are no warning labels about the potential of exposure to harmful products other than nicotine. E-liquids did not come under FDA regulation until 2015, and there are no current recommendations on the maximum concentration of NNN or NNK that should be present in e-liquids. Therefore, it is unsurprising that there is a wider distribution of TSNA levels in e-liquids in comparison to the tobacco products, which are heavily regulated. It should be noted, however, the majority of the e-liquids tested in this study were purchased prior to the 2015 FDA regulation. The four e-liquids that were bought after 2015 (Cedar Resesrve American Red, German Liquids Gold Blend, Virginia White Tobacco, and Virginia White United States Mix), had lower levels (less than 3 ng/ml) of TSNAs present, but it would be beneficial to use the developed method for e-liquids made in 2020 or later.

## 5 Conclusion

An analytical method for detecting tobacco specific nitrosamines NNN and NNK using a molecularly imprinted polymer packed-HPLC column was established and validated. The MIP-packed columns were uniform with regards to retention time, peak area, calculated concentration, and accuracy. Column characterization showed slight asymmetry and peak tailing, and a wide distribution of theoretical plated with high variability (% RSD >20%). The non-uniformity of the theoretical plates is most likely due to the large particle size and wide particle size distribution of the MIP polymer. The developed analytical was able to successfully detect NNN and NNK in multiple nicotine and tobacco products and e-liquids at the same levels in methods that use traditional HPLC columns, and did not require sample preparation such as solid phase extraction. Further, the MIP column resulted in better retention and detection of the analytes of interest when compared with a traditional C18 column under similar conditions. MIP-packed columns can allow for the direct, targeted analysis of analytes beyond consumer products (i.e., in waste water, biological samples, etc.,) with little benchtop preparation prior to instrumental analysis. The use of the MIP HPLC packing method can be utilized for in-line MIP protocols for other analytes routinely analyzed in bioanalytical methods.

## Supplementary Material

Direct analysis of tobacco specific nitrosamines in tobacco products using a molecularly imprinted poupplementary Figure S1 ∣ Nicotine and Tobacco products analyzed in this study: (A) 14 e-liquids; (B) 8 oral nicotine pouches and 3 Camel SNUS products; (C) one pipe tobacco product labelled as “dohka”. SUPPLEMENTARY FIGURE S1
Nicotine and Tobacco products analyzed in this study: **(A)** 14 e-liquids; **(B)** 8 oral nicotine pouches and 3 Camel SNUS products; **(C)** one pipe tobacco product labelled as “dohka”.

Direct analysis of tobacco specific nitrosamines in tobacco products using a molecularly imprinSupplementary Figure S2 ∣ Autosampler stability for NNN in 10 mM ammonium acetate, pH 5.5 and 70:30 PG:VG. Samples were kept on the autosampler, chilled to 5°C, and injected at 24 h intervals in triplicate over the course of 72 h. Stability was determined if sample concentrations were within ± 15% of the nominal value and calculated accuracy and had %RSD values < 15%. SUPPLEMENTARY FIGURE S2
Autosampler stability for NNN in 10 mM ammonium acetate, pH 5.5 and 70:30 PG:VG. Samples were kept on the autosampler, chilled to 5°C, and injected at 24 h intervals in triplicate over the course of 72 h. Stability was determined if sample concentrations were within ± 15% of the nominal value and calculated accuracy and had %RSD values < 15%.

Supplementary Figure S3 ∣ Autosampler stability for NNN in 10 mM ammonium acetate, pH 5.5 and 70:30 PG:VG. Samples were kept on the autosampler, chilled to 5°C, and injected at 24 h intervals in triplicate over the course of 72 h. Stability was determined if sample concentrations were within ± 15% of the nominal value and calculated accuracy and had %RSD values < 15%. SUPPLEMENTARY FIGURE S3
Autosampler stability for NNN in 10 mM ammonium acetate, pH 5.5 and 70:30 PG:VG. Samples were kept on the autosampler, chilled to 5°C, and injected at 24 h intervals in triplicate over the course of 72 h. Stability was determined if sample concentrations were within ± 15% of the nominal value and calculated accuracy and had %RSD values < 15%.

Direct analysis of tobacco specific nitrosamines in tobacco products using a molecularly imprinted Supplementary Figure S4 ∣ Representative chromatograph of 0.1 ng/mL NNK and the internal standard, NNN-d4 using a reverse phase C18 chromatographic column under similar analytical conditions. Column: Zorbax XDB C18 (2.1 × 50 mm, 5 μm); Isocratic gradient of 80:20 (v/v) 2 mM ammonium acetate in water: acetonitrile, flow rate of 0.45 mL/min. SUPPLEMENTARY FIGURE S4
Representative chromatograph of 0.1 ng/mL NNK and the internal standard, NNN-d4 using a reverse phase C18 chromatographic column under similar analytical conditions. Column: Zorbax XDB C18 (2.1 × 50 mm, 5 μm); Isocratic gradient of 80:20 (v/v) 2 mMammonium acetate in water: acetonitrile, flow rate of 0.45 mL/min.

## Figures and Tables

**TABLE 1:** Gradient conditions for the SCIEX Selexion LC 2.0 HPLC Pumps.

Time	Mobile phase A (%)	Mobile phase B (%)
0.0	100	0
1.0	40	60
2.0	40	60
3.0	0	100
3.1	0	100
4.0	0	100
4.1	100	0
5.0	100	0

a) Mobile Phase A: 10 mM ammonium acetate, pH 5.5.

b) Mobile Phase B: .1% formic acid in methanol.

**TABLE 2:** Accuracy and precision of quality control calibrators (*N* = 9) in nicotine and tobacco products (10 mM ammonium acetate, pH 5.5) and e-cigarette e-liquids (70:30 PG:VG). Concentration (ng/ml) is expressed as average ±standard deviation.

10 mM ammonium acetate, pH 5.5 (*N* = 9)						
Calibrator	NNN			NNk		
	Concentration (ng/ml)	Accuracy (%)	Precision (%)	Concentration (ng/ml)	Accuracy (%)	Precision (%)
LLQC (.1 ng/ml)	.09 ± .01	95.8	6.2	.10 ± .01	99.2	6.0
LQC (.3 ng/ml)	.27 ± .01	91.8	2.1	.27 ± .01	86.5	4.2
MQC (3.0 ng/ml)	2.90 ± .05	96.5	1.8	2.70 ± .07	88.1	2.4
HQC (7.5 ng/ml)	7.38 ± .12	97.8	1.6	7.05 ± .03	86.4	0.5
70:30 PG:VG (*N* = 9)						
Calibrator	NNN			NNk		
	Concentration (ng/ml)	Accuracy (%)	Precision (%)	Concentration (ng/ml)	Accuracy (%)	Precision (%)
LQC (.3 ng/ml)	.31 ± .04	102.6	14.1	.31 ± .01	100.6	3.7
MQC (3.0 ng/ml)	3.01 ± .14	100.5	4.9	3.08 ± .24	101.3	7.8
HQC (7.5 ng/ml)	7.80 ± .20	103.9	2.5	8.40 ± .19	109.7	2.3

**TABLE 3:** Column uniformity and characterization results for NNN and NNK with the three MIP-packed HPLC columns expressed as average (%RSD) for each parameter (*N* = 9).

10 mM ammonium acetate, pH 5.5 (*N* = 9)					
	Calibrator	Retention time (min)	Peak area	Concentration (ng/ml)	Accuracy (%)
NNN	LLQC (.1 ng/ml)	1.64 (1.2%)	19028 (8.7%)	.11 (5.6%)	106 (5.4%)
	LQC (.3 ng/ml)	1.64 (1.0%)	48559 (5.1%)	.27 (6.4%)	92 (6.4%)
	MQC (3.0 ng/ml)	1.64 (1.1%)	475267 (4.1%)	2.74 (2.4%)	91 (2.4%)
	HQC (7.5 ng/ml)	1.64 (1.1%)	1397370 (3.6%)	7.62 (7.9%)	101 (7.9%)
NNK	LLQC (.1 ng/ml)	1.75 (.8%)	23425 (8.2%)	.10 (13.3%)	95 (13.3%)
	LQC (.3 ng/ml)	1.76 (.8%)	53407 (4.0%)	.26 (9.5%)	87 (9.6%)
	MQC (3.0 ng/ml)	1.76 (.8%)	515287 (2.3%)	2.74 (3.3%)	91 (3.3%)
	HQC (7.5 ng/ml)	1.76 (.8%)	1526294 (1.4%)	8.35 (2.1%)	111 (2.1%)
70:30 PG:VG (*N* = 9)					
	Calibrator	Retention Time (min)	Peak Area	Concentration (ng/ml)	Accuracy (%)
NNN	LQC (.3 ng/ml)	1.64 (.7%)	60515 (11.9%)	.32 (4.9%)	107 (4.9%)
	MQC (3.0 ng/ml)	1.63 (.5%)	614715 (6.0%)	2.89 (3.2%)	96 (3.2%)
	HQC (7.5 ng/ml)	1.64 (.5%)	1417081 (9.5%)	7.49 (2.1%)	99 (2.1%)
NNK	LQC (.3 ng/ml)	1.76 (.7%)	74677 (18.4%)	.30 (5.5%)	119 (5.5%)
	MQC (3.0 ng/ml)	1.75 (.5%)	673063 (8.1%)	3.01 (4.5%)	100 (4.5%)
	HQC (7.5 ng/ml)	1.76 (.5%)	1631529 (8.1%)	7.91 (5.5%)	105 (5.5%)

**TABLE 4:** Column uniformity for the three MIP-packed HPLC columns expressed as range (%RSD) in 10 mM ammonium acetate, pH 5.5 (*N* = 36) and 70:30 PG: VG (*N* = 27).

	Asymmetry	Tailing factor	Theoretical plate number
10 mM ammonium acetate, pH 5.5 (*N* = 36)			
NNN	.9–1.7 (13.5%)	1.0–1.6 (12.9%)	90-304 (28.3%)
NNK	1.0–1.7 (12.3%)	1.0–1.5 (8.3%)	108-317 (28.2%)
70:30 PG:VG (*N* = 27)			
NNN	.9–1.4 (13.2%)	1.0–1.2 (6.7%)	128-311 (29.4%)
NNK	1.1–2.2 (20.9%)	1.1–2.2 (20.9%)	103-297 (32.5%)

**TABLE 5:** TSNA levels in nicotine and tobacco products.

Sample results			
Sample	Type	NNN (ng/mL or μg/g)	NNK (ng/mL or μg/g)
Avail Captain’s Cut	E-liquid	28 ± 2	53 ± 5
Avail Continental Breakfast	E-liquid	BDL	1.8 ± .07
Avail Sapphire Morning	E-liquid	203 ± 6	35.7 ± 0.9
Avail Seduction	E-liquid	94 ± 33	17.1 ± 5.3
Cedar Reserve American Red	E-liquid	BDL	BDL
German Liquids Golden Blend	E-liquid	BDL	BDL
High Voltage Melatonin	E-liquid	BDL	BDL
Nirvana Citrus OD	E-liquid	BDL	1.6 ± 0.2
Nirvana Headrush	E-liquid	BDL	38.2 ± 2.3
Palm Strawberry Flavor	E-liquid	29 ± 2	BDL
Supreme Nicotine 258 Rally Squirrel	E-liquid	5.9 ± 0.4	21.3 ± 1.6
Top Vapor Honeydew	E-liquid	21 ± 1	19.3 ± 0.5
Virginia White United States of America Mix	E-liquid	2.1 ± 0.2	BDL
Virginia White Tobacco	E-liquid	1.5 ± 0.1	BDL
Camel Mellow	SNUS	1.61 ± .02	.454 ± .080
Camel Mint	SNUS	1.37 ± .05	.441 ± .040
Camel SNUS Mint	SNUS	1.49 ± .04	.455 ± .028
Nirvana Skull Control	Pipe Tobacco	1.87 ± .04	BDL

a) *N* = 3 for each sample injection.

b) BDL, below detection limit.

c) No TSNAs, were detected in the eight oral nicotine pouches.

## Data Availability

The original contributions presented in the study are included in the article/Supplementary Material, further inquiries can be directed to the corresponding author.
