# Supplementary figures and images for: Direct analysis of tobacco specific nitrosamines in tobacco products using a molecularly imprinted polymer-packed column
Source: Front Anal Sci. Author manuscript; Available in PMC 2024 Jan 6. (PMC10540244; doi:10.3389/frans.2022.1091206)

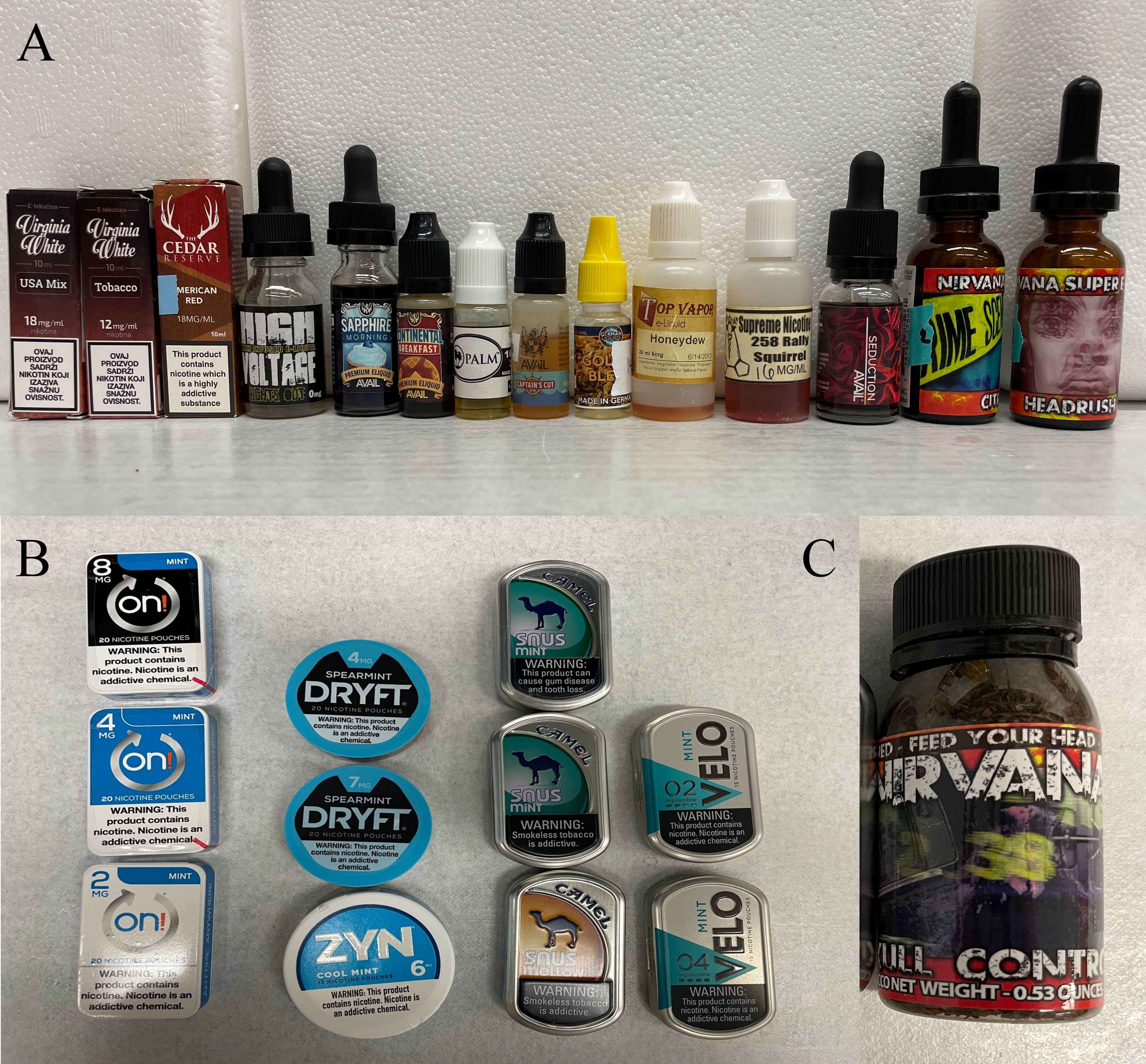

Supplement: Direct analysis of tobacco specific nitrosamines in tobacco products using a molecularly imprinted poupplementary Figure S1 ∣ Nicotine and Tobacco products analyzed in this study: (A) 14 e-liquids; (B) 8 oral nicotine pouches and 3 Camel SNUS products; (C) one pipe tobacco product labelled as “dohka — SUPPLEMENTARY FIGURE S1 Nicotine and Tobacco products analyzed in this study: (A) 14 e-liquids; (B) 8 oral nicotine pouches and 3 Camel SNUS products; (C) one pipe tobacco product labelled as “dohka”. [file NIHMS1930970-supplement-Direct_analysis_of_tobacco_specific_nitrosamines_in_tobacco_products_using_a_molecularly_imprinted_poupplementary_Figure_S1___Nicotine_and_Tobacco_products_analyzed_in_this_study___A__14_e-liquids___B__8_oral_nicotine_.tiff]

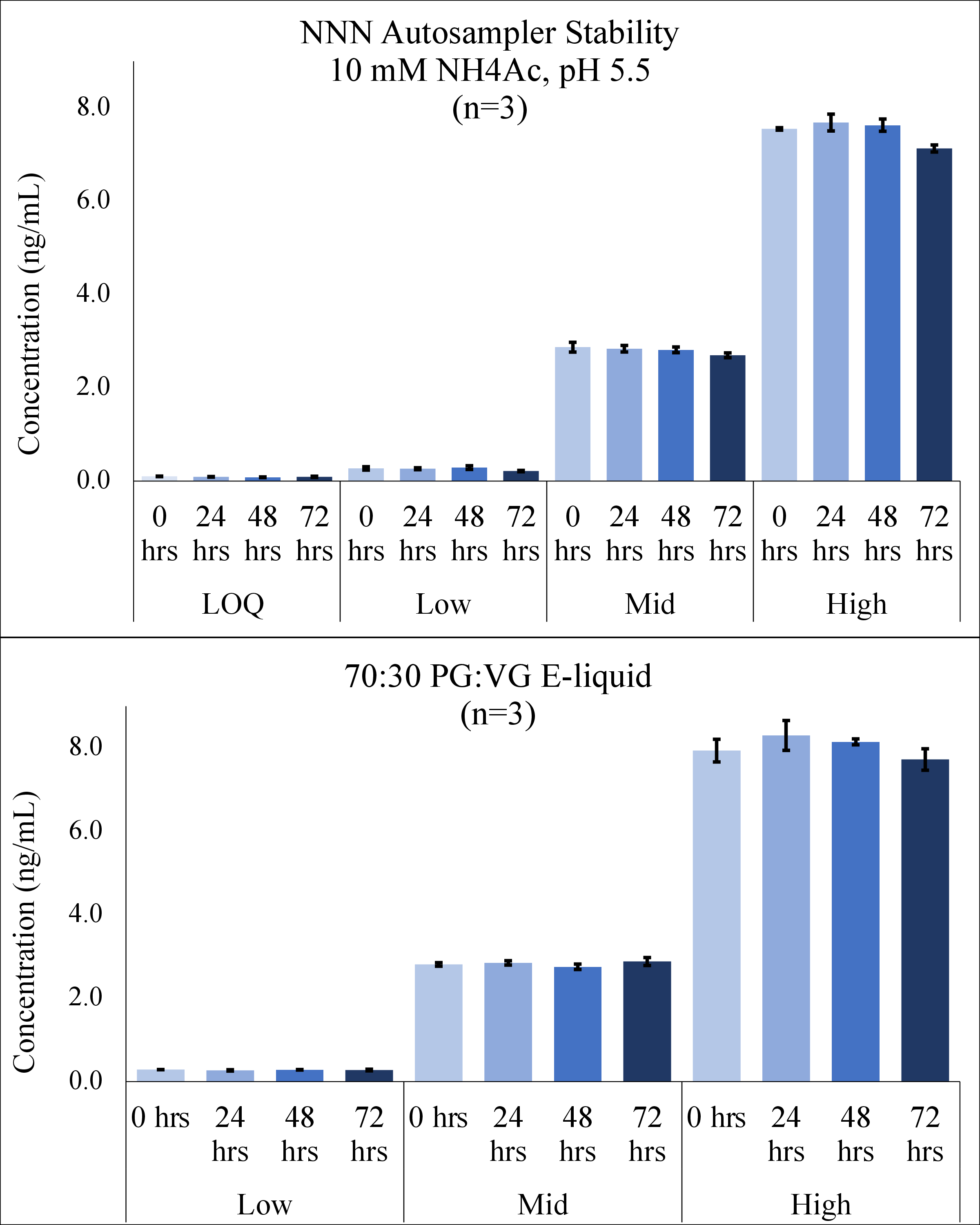

Supplement: Direct analysis of tobacco specific nitrosamines in tobacco products using a molecularly imprinSupplementary Figure S2 ∣ Autosampler stability for NNN in 10 mM ammonium acetate, pH 5.5 and 70:30 PG:VG. Samples were kept on the autosampler, chilled to 5°C, and injected at 24 h intervals in triplicate — SUPPLEMENTARY FIGURE S2 Autosampler stability for NNN in 10 mM ammonium acetate, pH 5.5 and 70:30 PG:VG. Samples were kept on the autosampler, chilled to 5°C, and injected at 24 h intervals in triplicate over the course of 72 h. Stability was determined if sample concentrations were within ± 15% of the nominal value and calculated accuracy and had %RSD values < 15%. [file NIHMS1930970-supplement-Direct_analysis_of_tobacco_specific_nitrosamines_in_tobacco_products_using_a_molecularly_imprinSupplementary_Figure_S2___Autosampler_stability_for_NNN_in_10_mM_ammonium_acetate__pH_5_5_and_70_30_PG_VG__Samples_were_kep.tiff]

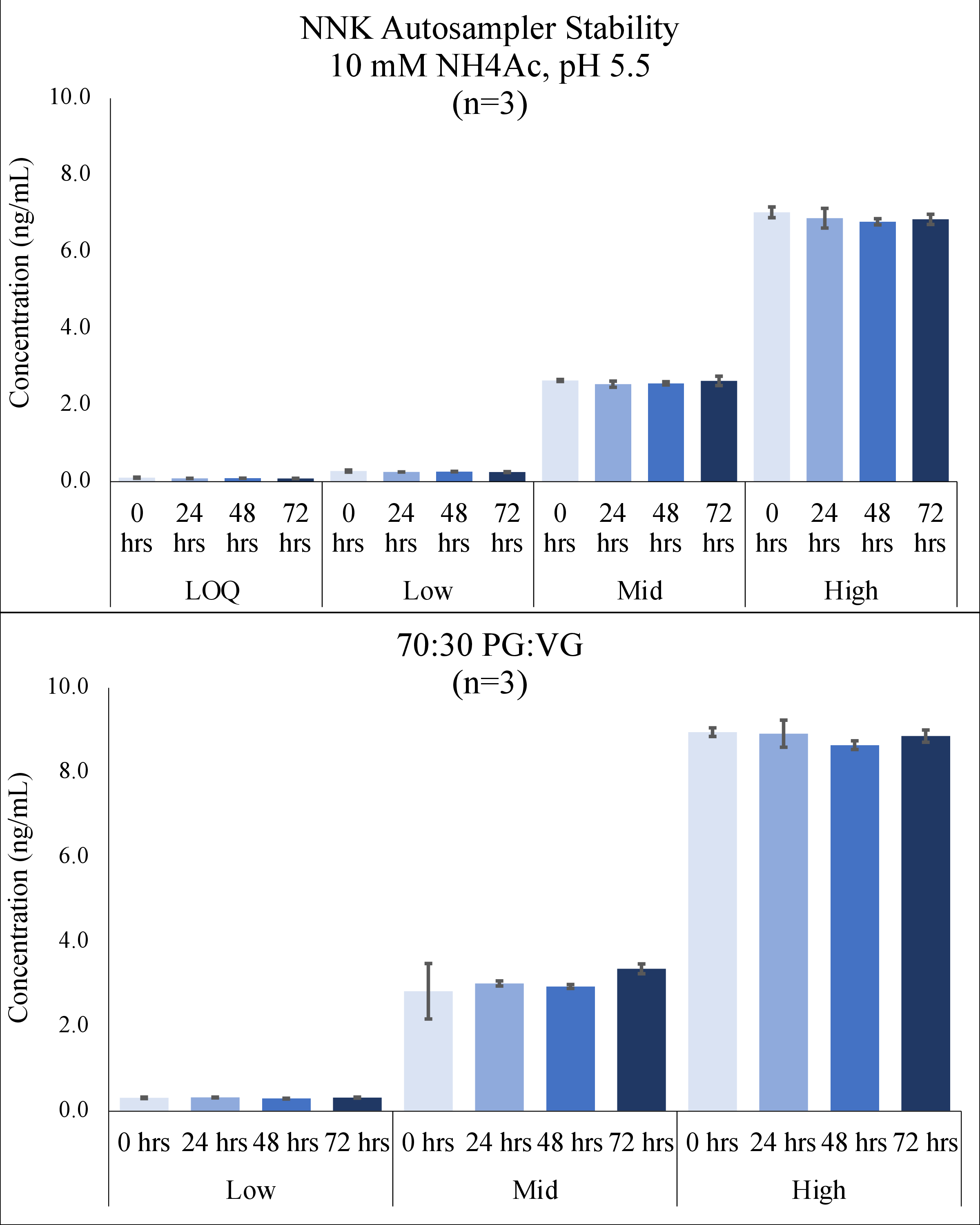

Supplement: Supplementary Figure S3 ∣ Autosampler stability for NNN in 10 mM ammonium acetate, pH 5.5 and 70:30 PG:VG. Samples were kept on the autosampler, chilled to 5°C, and injected at 24 h intervals in triplicate over the course of 72 h. Stability was determined if sample concentrations were within ± 15% o — SUPPLEMENTARY FIGURE S3 Autosampler stability for NNN in 10 mM ammonium acetate, pH 5.5 and 70:30 PG:VG. Samples were kept on the autosampler, chilled to 5°C, and injected at 24 h intervals in triplicate over the course of 72 h. Stability was determined if sample concentrations were within ± 15% of the nominal value and calculated accuracy and had %RSD values < 15%. [file NIHMS1930970-supplement-Supplementary_Figure_S3___Autosampler_stability_for_NNN_in_10_mM_ammonium_acetate__pH_5_5_and_70_30_PG_VG__Samples_were_kept_on_the_autosampler__chilled_to_5_C__and_injected_at_24_h_intervals_in_triplicate_over_the_cou.tiff]

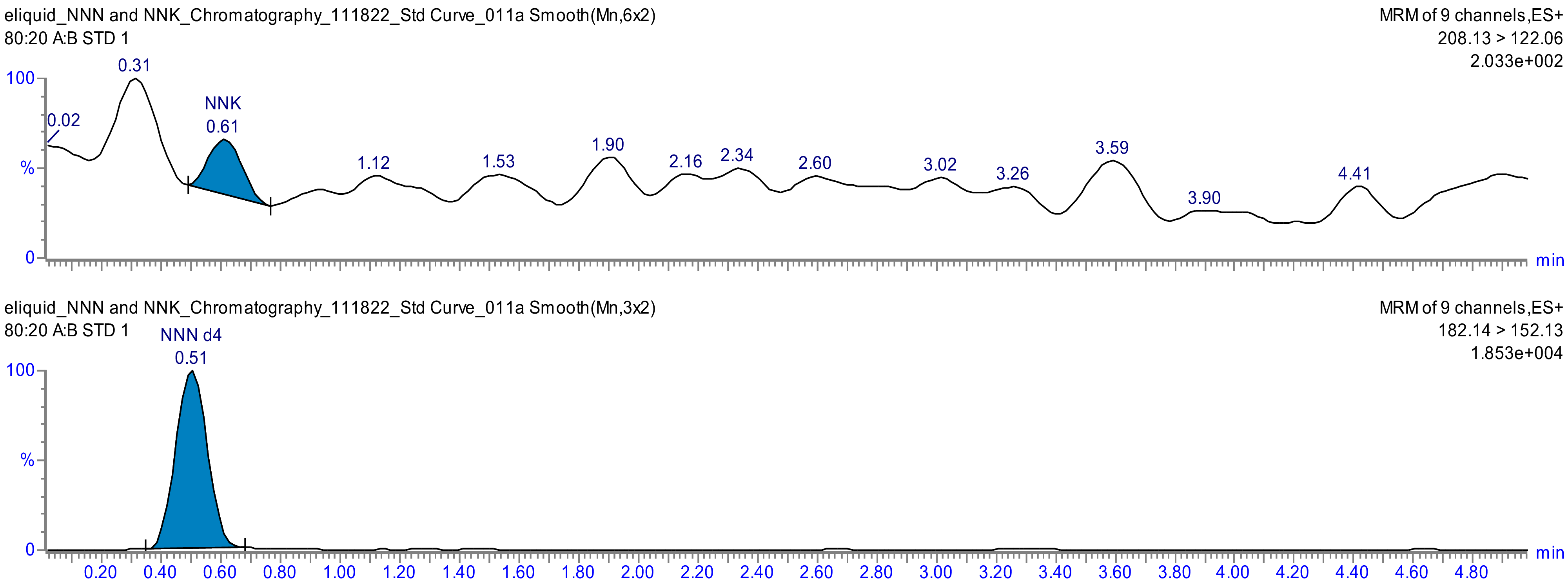

Supplement: Direct analysis of tobacco specific nitrosamines in tobacco products using a molecularly imprinted Supplementary Figure S4 ∣ Representative chromatograph of 0.1 ng/mL NNK and the internal standard, NNN-d4 using a reverse phase C18 chromatographic column under similar analytical conditions. Column: Z — SUPPLEMENTARY FIGURE S4 Representative chromatograph of 0.1 ng/mL NNK and the internal standard, NNN-d4 using a reverse phase C18 chromatographic column under similar analytical conditions. Column: Zorbax XDB C18 (2.1 × 50 mm, 5 μm); Isocratic gradient of 80:20 (v/v) 2 mMammonium acetate in water: acetonitrile, flow rate of 0.45 mL/min. [file NIHMS1930970-supplement-Direct_analysis_of_tobacco_specific_nitrosamines_in_tobacco_products_using_a_molecularly_imprinted_Supplementary_Figure_S4___Representative_chromatograph_of_0_1_ng_mL_NNK_and_the_internal_standard__NNN-d4_using_a_rever.tiff]
